# Bilateral Wrist Drop Caused by Non-invasive Continuous Monitoring of Blood Pressure During Severe COVID-19 Pneumonia: A Case Report

**DOI:** 10.7759/cureus.22212

**Published:** 2022-02-14

**Authors:** Busra S Arica Polat, Kubra Isik, Tugce Mengi, Ersin Tan, Zeki Odabasi

**Affiliations:** 1 Neurology, Gulhane Training and Research Hospital, Ankara, TUR; 2 Neurology, Nigde Training and Research Hospital, Nigde, TUR; 3 Neurology, Hacettepe University, Ankara, TUR; 4 Neurology, Gulhane Medical School, Ankara, TUR

**Keywords:** icu, radial nerve injury, covid-19 pneumonia, compressive neuropathy, wrist drop

## Abstract

Compressive peripheral nerve injury can be observed as a long-term outcome during the treatment of severe COVID-19 pneumonia. In this case study, we report a man with bilateral wrist drop due to prolonged noninvasive blood pressure monitoring. A 52-year-old man who had undergone invasive ventilation because of severe COVID-19 pneumonia was admitted with bilateral loss of function of the wrist, digital, and thumb extensors and hypoesthesia in the dorsum of the forearm and hand. The patient had not been treated with prone positioning respiratory therapy. However, he had undergone bilateral automated sphygmomanometry that measured his blood pressure every ten minutes during his ICU stay. His electrophysiological findings were compatible with the presence of bilateral radial nerve compression at the level of the spiral groove. Awareness of potential compressive peripheral nerve injury is important for rehabilitation after the treatment of COVID-19-associated pneumonia.

## Introduction

Coronavirus disease-19 (COVID-19) varies from asymptomatic or mild symptomatic respiratory illness to severe pneumonia. Severe respiratory syndrome leads to the patient requiring intensive care unit (ICU) admission and results in prolonged hospitalization [1]. As the number of survivors increases after effective treatment of COVID-19 infection, there is an increase in sequelae related to long-term neuromuscular pathological conditions due to long-term hospitalization and patients’ positioning in the ICU [2]. As a result of these situations, rehabilitation needs become more important [3]. Although compressive peripheral nerve injury can be observed as a long-term outcome during the care of COVID-19 patients, bilateral radial nerve paralysis has not been reported previously [4]. In the present case study, we report a man with bilateral radial nerve paralysis due to automated noninvasive continuous monitoring of blood pressure during severe COVID-19 pneumonia.

## Case presentation

A 52-year-old man, suffering from bilateral loss of function in the wrist, digital, and thumb extensors and hypoesthesia in the dorsum of the forearm and hand was admitted to the neurology department. The patient had been diagnosed with severe COVID-19 pneumonia about four months previously and invasive ventilation (IV) had been applied for 17 days in the ICU. He had received intensive antibiotic treatment due to secondary bacterial infections in addition to treatment with favipiravir (8 g for 10 days), plasma exchange, and cytokine filter. The patient had not been treated with prone positioning respiratory therapy and central venous catheterization had not been applied. However, he had undergone bilateral automated sphygmomanometry that measured his blood pressure every ten minutes during his ICU stay.

**Figure 1:**
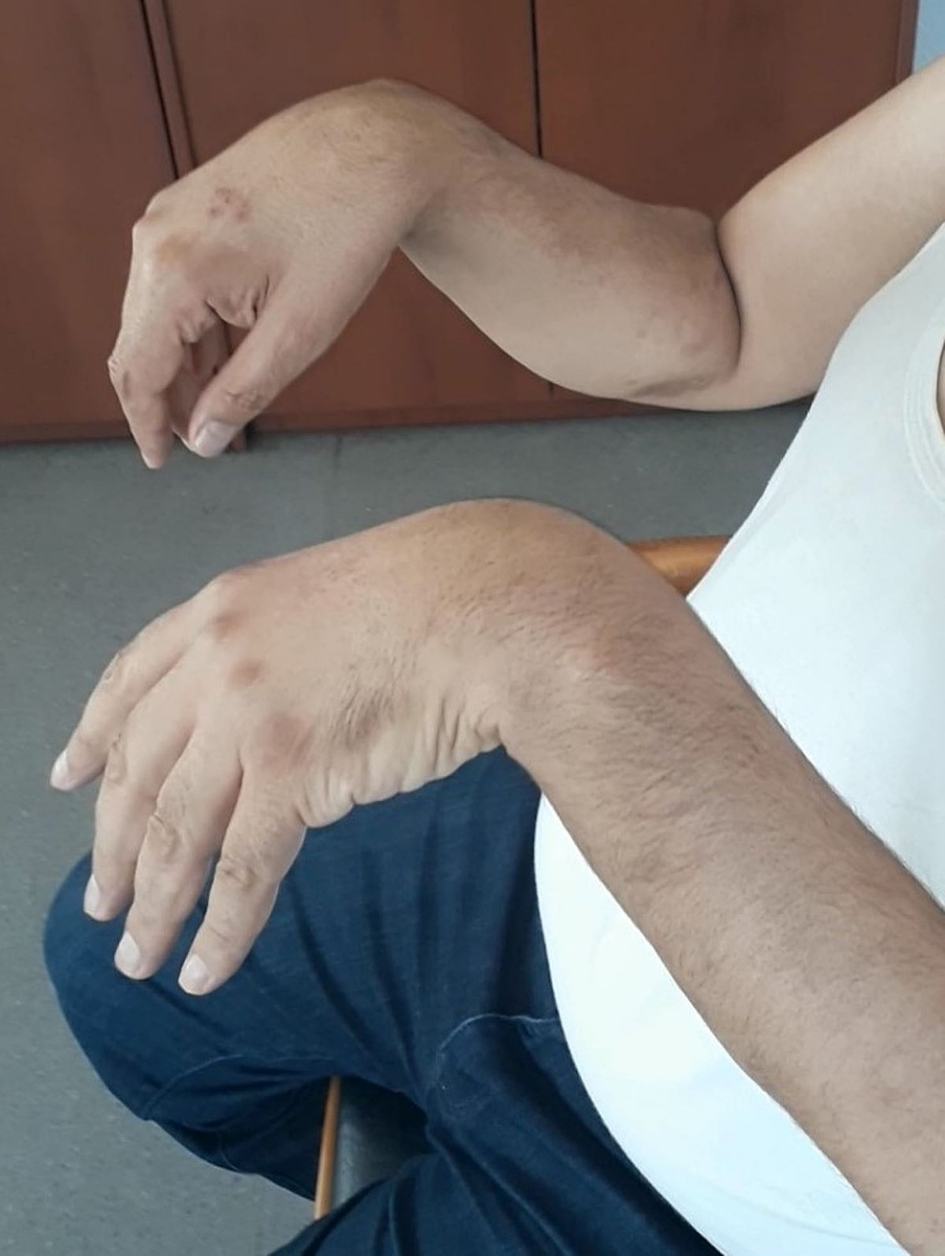
Bilateral wrist drop; loss of muscular strength in extensors of bilateral wrist and digits.

After pulmonary distress resolution and ceasing sedation, weakness of the extensor muscles of the elbow and hypoesthesia of the hand were observed bilaterally. These complaints continued without improvements following discharge from the hospital. His neurological examination showed loss of muscular strength in the extensors of the wrist and digits bilaterally, inability to extend the limb (called wrist drop), and hypoesthesia in the area of the dorsum of the hand innervated by the radial nerve (Figure 1). No other risk factors for compressive neuropathy were identified. There were no sensory nerve action potentials in the superficial radial nerve and no compound muscle action potentials in the radial nerve recorded in the extensor indicis proprius muscles bilaterally. Nerve conduction studies were normal in the bilateral median, ulnar, medial, and lateral antebrachial cutaneous nerves. In the needle examination, there was total denervation in the bilateral extensor indicis proprius, extensor digitorum communis, and brachioradialis. The triceps, deltoids, and biceps were normal according to the needle examination. These electrophysiological findings were compatible with the presence of bilateral radial nerve compression at the level of the spiral groove due to prolonged noninvasive blood pressure monitoring. The patient was referred for rehabilitation.

## Discussion

Compressive peripheral nerve injury in COVID-19 patients can occur as a complication of treatment for severe pneumonia. It may result from traction and/or pressure injury during lifting, repositioning, and prolonged prone positioning of patients [5,6]. The ulnar, radial, and median nerves and brachial plexus in the upper limb and the sciatic nerve in the lower limb have been reported as the most frequent sites of injury as sequelae of the prolonged prone positioning in COVID-19-related respiratory distress syndrome [6]. The incidence of peripheral nerve injury during the care of COVID-19 patients other than prone positioning is unknown. Otherwise, bilateral radial paralysis during hospitalization of a patient with COVID-19 has not been reported before. To the best of our knowledge, our case is the first of bilateral radial paralysis most likely induced by a blood pressure cuff in a COVID-19 patient.

It has been identified that compression of the radial nerve can occur due to external equipment, such as blood pressure cuffs, along the posterior aspect of the arm at the level of the spiral groove in intraoperative positioning [7]. The extensor muscle weakness and sensory deficit of the wrist and digits in our case were determined following frequent and prolonged blood pressure monitoring by bilateral cuffs during treatment for COVID-19 pneumonia. Other etiological factors that could cause peripheral neuropathy (i.e., diabetes, smoking history, and alcoholism) were ruled out. Unlike other cases in the literature showing peripheral nerve injury associated with prone positioning, our patient was not treated in this position during his hospitalization. The pressure on the radial nerve while in the side position by arm rest or arm board or other objects (a common cause for perioperative radial nerve palsy) can be another possible factor for radial nerve palsy [8]. These other possibilities for radial nerve palsy were questioned. However, any possibilities were not available in the presented patient. Therefore, the clinical manifestation of the patient can only be explained by the bilateral noninvasive continuous monitoring of his blood pressure.

After recovery from COVID-19-associated pneumonia, positioning and compressive peripheral nerve injury are important in terms of rehabilitation [9]. Consequently, attention to potential nerve compression and stretching while life-saving measures are being implemented during the care of COVID-19 patients may reduce the likelihood of peripheral nerve injury [10].

## Conclusions

Compressive peripheral nerve injury is already a known condition; however, this case study reports the first patient to present with bilateral radial nerve paralysis most likely due to prolonged frequent sphygmomanometry during treatment for severe COVID-19 pneumonia in the ICU.
